# Increased risk for autochthonous vector-borne infections transmitted by *Aedes albopictus* in continental Europe

**DOI:** 10.2807/1560-7917.ES.2018.23.24.1800268

**Published:** 2018-06-14

**Authors:** Céline M Gossner, Els Ducheyne, Francis Schaffner

**Affiliations:** 1 European Centre for Disease Prevention and Control, Solna, Sweden; 2 Avia-GIS, Zoersel, Belgium; 3 Francis Schaffner Consultancy, Riehen, Switzerland; 4 National Centre for Vector Entomology, Institute of Parasitology, Vetsuisse Faculty, University of Zurich, Zurich, Switzerland

**Keywords:** Aedes albopictus, Outbreak, Europe, vector-borne diseases, dengue, chikungunya, emerging diseases, arboviruses

## Abstract

Autochthonous outbreaks of chikungunya and dengue during the past decade showed that continental Europe is vulnerable to *Aedes albopictus*–borne infections. *Ae. albopictus* has spread geographically, resulting in more people exposed to risk. Timely application of adequate mosquito suppression measures may delay, or even prevent, the vector population from crossing the potential epidemic abundance threshold should a pathogen be introduced. Health authorities should be on alert to detect early cases to prevent autochthonous outbreaks.

*Aedes albopictus*, commonly called the Asian tiger mosquito, is an invasive mosquito species that is rapidly spreading across Europe [1]. With the start of the mosquito activity season, we assess the risk of geographical spread of *Ae. albopictus* to public health for the coming and successive mosquito seasons in continental Europe.

## Risk of autochthonous disease outbreaks in Europe

*Ae. albopictus* is a known vector of chikungunya and dengue viruses, and experimental studies have indicated potential for this mosquito species to also be a vector of, among other, Japanese encephalitis and West Nile viruses [2].

The first autochthonous chikungunya outbreak in continental Europe, with *Ae. albopictus* as vector was reported in 2007 from north-eastern Italy, resulting in more than 200 laboratory–confirmed cases [2]. Since then, mainland France has reported three chikungunya outbreaks: two in the Var department (in 2010 and 2017, respectively) and one in the Hérault department in 2014 [2-4]. Italy reported an outbreak in the Lazio and Calabria regions in 2017 with over 400 cases [5]. Since 2010, seven autochthonous dengue outbreaks have been reported in continental Europe, six in France (2010, 2013, 2014 (three outbreaks) and 2015) and one in Croatia (2010) [2,6-9]. These outbreaks followed the introduction of chikungunya and dengue viruses by viraemic travellers returning from areas where the viruses are known to circulate. They all occurred in areas where *Ae. albopictus* is established and at a time when the environmental conditions in Europe were favourable to vector activity and virus replication i.e. between July and October for chikungunya and between August and October for dengue.

## Distribution and spread of the vector

Under the 2014–2018 VectorNet project, the European Centre for Disease Prevention and Control (ECDC) and the European Food Safety Authority (EFSA) have collected distribution data on *Ae. albopictus* in Europe and neighbouring countries [10]. Every quarter, distribution maps have been published on the ECDC website.

**Figure 1:**
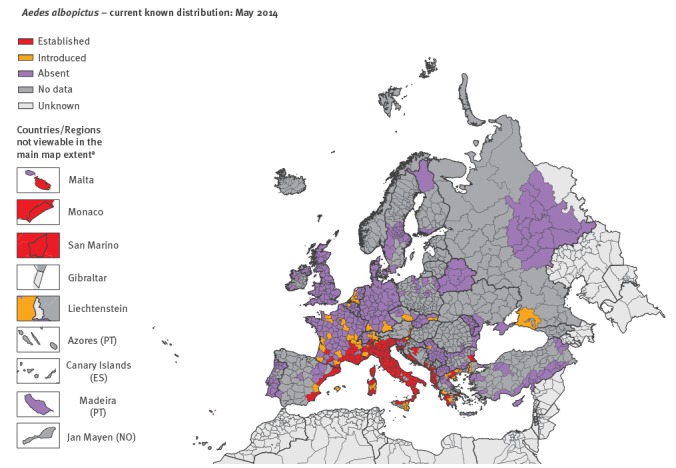
Distribution of *Aedes albopictus* in Europe and neighbouring countries, May 2014

^a^Countries/Regions are displayed at different scales to facilitate their visualisation.

**Figure 2:**
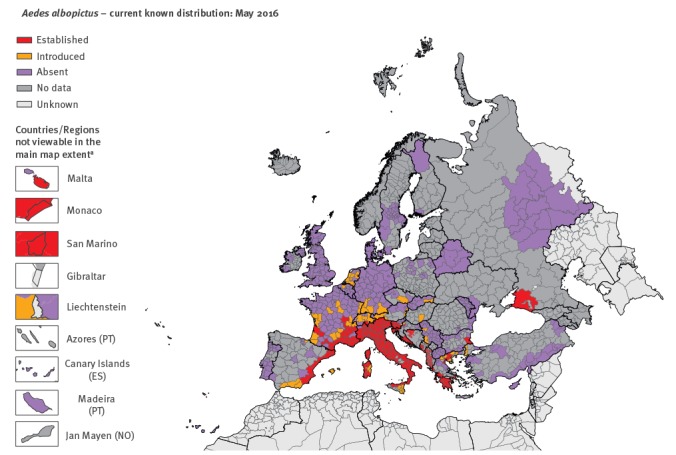
Distribution of *Aedes albopictus* in Europe and neighbouring countries, May 2016

^a^Countries/Regions are displayed at different scales to facilitate their visualisation.

**Figure 3:**
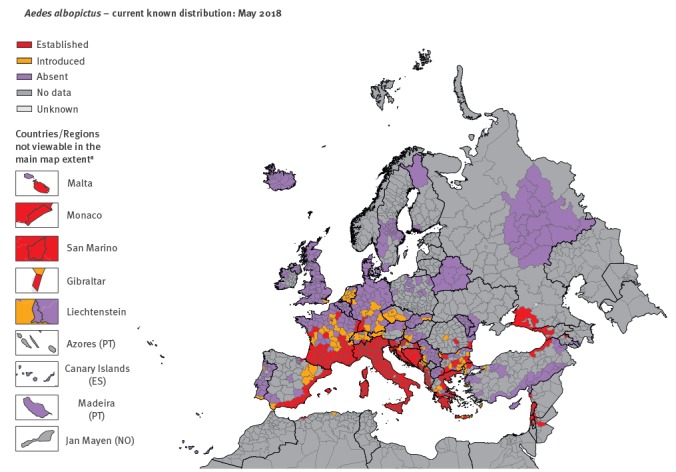
Distribution of *Aedes albopictus* in Europe and neighbouring countries, May 2018

^a^Countries/Regions are displayed at different scales to facilitate their visualisation.

Results from the VectorNet surveillance showed a rapid spread of *Ae. albopictus* from long-term invaded areas to new areas. The number of areas considered as having the mosquito ‘introduced’ and ‘established’ at the third administrative level of the Nomenclature of Territorial Units for Statistics (NUTS-3) or similar units in countries not covered by the NUTS, have increased over the years (Figure 1, 2, 3). For a mapping unit to obtain an ‘established’ status, the mosquito population has to show evidence of local reproduction and overwintering.

The distribution map shows ‘introduced’ and ‘established’ statuses at the third administrative level of the Nomenclature of Territorial Units for Statistics (NUTS-3) or similar units in countries not covered by the NUTS. For a mapping unit to obtain an ‘established’ status, the mosquito population has to show evidence of local reproduction and overwintering.

Map produced on 11 Jun 2018. Data presented in this map is collected through the VectorNet project.

The distribution map shows ‘introduced’ and ‘established’ statuses at the third administrative level of the Nomenclature of Territorial Units for Statistics (NUTS-3) or similar units in countries not covered by the NUTS. For a mapping unit to obtain an ‘established’ status, the mosquito population has to show evidence of local reproduction and overwintering.

Map produced on 11 Jun 2018. Data presented in this map is collected through the VectorNet project.

The distribution map shows ‘introduced’ and ‘established’ statuses at the third administrative level of the Nomenclature of Territorial Units for Statistics (NUTS-3) or similar units in countries not covered by the NUTS. For a mapping unit to obtain an ‘established’ status, the mosquito population has to show evidence of local reproduction and overwintering.

Map produced on 11 Jun 2018. Data presented in this map is collected through the VectorNet project.

In few instances, new observations were related to the recent implementation of surveillance in countries such as Bosnia and Herzegovina or in eastern Turkey. In most of countries however, surveillance has been ongoing for several years, and findings of new mosquito populations therefore indicate spread.

*Ae. albopictus* has been long–established in most parts of southern Europe, with Albania and Italy being the first colonised countries in 1979 and 1990, respectively [1]. It was recently introduced in Portugal at both southern and north-eastern locations and in the United Kingdom (UK) (Figure 3). Recently *Ae. albopictus* has become established in new areas of Spain, Gibraltar, France, where the number of ‘introduced’ and ‘established’ administrative units have doubled over the last two years, and Baden-Württemberg in the south of Germany, Limburg province in the Netherlands, as well as in Bulgaria, Croatia, Romania and around the Black Sea eastern coast (Figure 3). It is predicted, based on risk–modelling, that *Ae. albopictus* has the potential to spread further north, as a consequence of progressive migration into areas that are suitable for the species to become established [11].

## Start of the vector activity season

In southernmost regions of Europe, *Ae. albopictus* females can remain active during winter time i.e. biting and egg-laying has been observed in Spain and Italy, and even larvae can survive the winter and develop slowly up to engender adult emergence [12-14]. However where this occurs, the population is scarce and has no public health relevance. Generally in Europe, the mosquito population only survives at the egg stage during winter and larvae will only start to develop when the egg diapause ends. This diapause termination is primarily induced by the increase in hours of daylight, while spring temperature influences the larval development duration and start of the adult vector activity season.

For example in the south of France, egg hatching in spring is usually observed around mid-March [15]. Given that this spring generation has a relatively long aquatic larval development phase, adult mosquitoes are expected to appear during the first half of May.

While the first adults produced by the overwintering eggs appear, adult population numbers are not expected to reach the potential epidemic abundance threshold until the end of June in the Mediterranean region. This suggests that the implementation of active mosquito suppression measures in May and June may delay, or even prevent, the vector population from crossing this threshold [16].

## Risk of introduction of viruses into Europe

### Chikungunya, dengue and Zika viruses

Chikungunya, dengue and Zika viruses are endemic/endemo-epidemic in large regions of the intertropical zone [17]. In the Americas, the circulation of the three viruses seems to be so far less extensive in 2018 than in previous years [18,19]. In Asia and in the Pacific region, no major chikungunya, dengue or Zika virus outbreaks have been detected to date in 2018 [19].

In Africa, the ongoing serotype 2 dengue outbreak in the French overseas department of Réunion is of concern as a potential source of importation to Europe resulting in a risk to public health. Between January and early June 2018, the French public health authorities reported 4,604 cases on Réunion [20]. So far, week on week, the number of cases reported has been increasing; cases are expected to occur until the start of the Austral winter in July. Therefore, the risk of importation of viraemic dengue cases from Réunion predominantly to mainland France and of subsequent autochthonous transmission is expected to be highest in the period from May to July. According to the International Air Transport Association (IATA), during that period 45,750 travellers will be returning from Réunion to Europe per month, with 98% destined for metropolitan France [21]. In this context, there is a risk of introduction of the virus into Europe, and particularly into mainland France.

In most dengue outbreaks, *Ae. aegypti* is the primary vector of infection, while *Ae. albopictus* is the primary vector in the outbreak on Réunion. This may suggest a virus-vector adaptation and hypothetically, adaptation of the virus to the *Ae. albopictus* populations of continental Europe [22]. This happened for chikungunya virus during the large outbreak on Réunion in 2006 [23]. Despite this cannot be directly transposed to dengue virus, this shows that the possibility exists. To date, in 2018, no autochthonous dengue outbreaks have been detected in mainland France; enhanced surveillance in regions where *Ae. albopictus* is established is crucial to detect virus introduction and possible local transmission.

### Yellow fever

Since December 2016, Brazil has faced a large outbreak of yellow fever with over 2,000 cases [24]. Since the beginning of 2018, 13 unvaccinated European travellers have contracted yellow fever in Brazil [25]. No new cases have been reported since 16 May 2018 from the epidemic area, similar to 2017, when the seasonal transmission of yellow fever virus in Brazil lasted until the end of May. This resulted in a short window of risk for viraemic travellers to introduce the virus into Europe during the period starting in May when *Ae. albopictus*, a vector competent in laboratory setting, is active and thus could possibly initiate an autochthonous transmission cycle [26].

## Discussion and conclusion

With the start of the vector adult activity season in Europe, health actors in areas where *Ae. albopictus* is established should be aware of the risk of introduction and subsequent possible autochthonous spread of arboviruses. There is a need for vigilance and increased awareness among local clinicians and among travellers returning from regions endemic for these viruses. This, combined with adequate laboratory diagnostic capability, is paramount for the early detection of travel-associated cases and crucial for the subsequent prevention of local transmission in areas where *Ae. albopictus* is established.

In order to detect possible resurgence of the virus after overwintering in the local mosquito population, virus surveillance should be strengthened in areas in Italy that were facing the large outbreak of chikungunya in 2017 [5]. Vertical transmission of chikungunya virus to offspring can occur and even if its epidemiological relevance is not evidenced, the pathogen surveillance may be implemented from the beginning of the season in these areas [27]. It is suggested that the detection of an autochthonous case should trigger epidemiological and entomological investigations to assess the risk of onward transmission and to guide focal vector control measures, according to a pre-defined national or local integrated surveillance and control plan [28]. Vector control measures must be applied rapidly if not immediately where the occurrence of the vector population is already known. Awareness of the risks associated with travel to disease-endemic regions should be highlighted to travellers and information on the symptoms of a possible mosquito-borne infection (e.g. fever and rash) should be provided.

*Aedes aegypti*, the main vector of dengue, chikungunya, Zika and yellow fever viruses around the world is, to date, only present in the most Eastern part of continental Europe, around the Black Sea [10]; the mosquito is also established in the European outermost region of Madeira as well as in many European overseas countries and territories. *Ae. aegypti* is colonising new areas and further spread would increase the overall risk of autochthonous transmission of *Aedes*-related diseases in continental Europe.
